# Lytic characterization and application of listerial endolysins PlyP40 and PlyPSA in queso fresco

**DOI:** 10.3168/jdsc.2020-0013

**Published:** 2021-01-22

**Authors:** Maxwell J. Holle, Michael J. Miller

**Affiliations:** Department of Food Science and Human Nutrition, University of Illinois at Urbana-Champaign, Urbana 61801

## Abstract

Queso fresco (QF) is a fresh Hispanic-style cheese that is commonly associated with the human foodborne pathogen *Listeria monocytogenes* and outbreaks of listeriosis in the United States. Endolysins, cell wall hydrolases derived from bacteriophages, are promising candidates for controlling bacterial pathogens in food systems. In this study, we characterized the lytic capabilities of 2 endolysins, PlyP40 and PlyPSA, under varying conditions (pH, temperature, salt concentration) and compared their activities with those of the previously described endolysin PlyP100. We showed that PlyP40 was effective, showing at least a 33% reduction in cellular debris, against a broader range of *Listeria* than PlyPSA, which showed little lytic activity toward *Listeria* strains not from serovar 4. Both endolysins were also capable of maintaining lytic activity to varying extents at refrigeration temperature. The effect of salt concentration and pH differed between PlyP40 and PlyPSA. Furthermore, we added the endolysins to QF and monitored their ability to control *L. monocytogenes* contamination over 28 d of cold storage. Both PlyP40 and PlyPSA were capable of lowering QF inoculum cell counts compared with the control; however, both were less effective than the previously characterized PlyP100. Further characterization of endolysins will continue to open opportunities to optimization and implementation in a variety of food matrices for controlling pathogen contamination.

## Highlights

• PlyP40 had lytic efficacy against a broad range of *Listeria*
• PlyP40 and PlyPSA were able to maintain lytic activity at refrigeration temperature
• Lytic activity of PlyP40 decreased as pH increased, whereas that of PlyPSA increased
• PlyP40 and PlyPSA maintained lytic activity within the queso fresco (QF) salt range
• PlyP40 and PlyPSA were able to decrease counts of *Listeria monocytogenes* in QF

## Graphical Abstract

**Figure:**
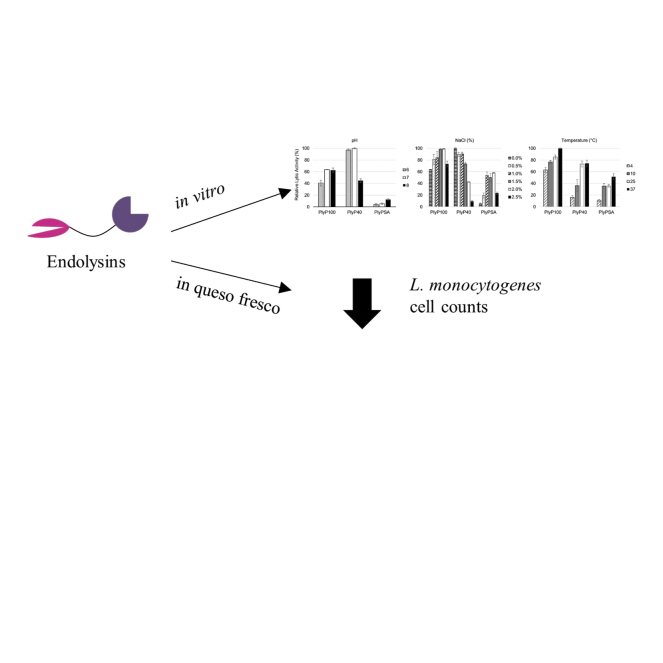
**Summary:** Endolysins, cell wall hydrolases derived from bacteriophages, are promising candidates for controlling bacterial pathogens in food systems; however, only a few endolysins have had their lytic capabilities characterized. In this study, changes in lytic activity against *Listeria* for endolysins PlyP40 and PlyPSA were monitored under varying environmental conditions in vitro and compared with the previously studied endolysin PlyP100. Additionally, the individual endolysins were added to queso fresco (QF) contaminated with *Listeria monocytogenes*. PlyP40 was able to lower the inoculum cell count over the 28-d shelf life of QF, and PlyPSA was able to lower the cell count until d 14. However, neither endolysin was better than PlyP100 at controlling *Listeria monocytogenes* in QF.

*Listeria monocytogenes* is a ubiquitous gram-positive and psychrotrophic pathogen. Human consumption of food contaminated by *L. monocytogenes* can lead to listeriosis, especially in at-risk populations such as infants, pregnant women, the elderly, and the immunocompromised. Although *L. monocytogenes* is a zero-tolerance organism in ready-to-eat foods, dairy products are highly associated with *L. monocytogenes* contamination in the United States (Batz et al., 2011). Despite most cheeses not being associated with pathogen contamination, Hispanic-style fresh cheeses are frequently contaminated with *L. monocytogenes* (Ibarra-Sánchez et al., 2017). Almost 20% of US-reported listeriosis outbreaks between 1998 and 2014 implicated Hispanic-style cheeses, which accounted for 54% of the total listeriosis cases during this timeframe (Jackson et al., 2018). Queso fresco (**QF**), the most widely produced fresh Hispanic-style cheese in the United States (Ibarra-Sánchez et al., 2018), is frequently implicated in listeriosis outbreaks (CDC, 2017, Jackson et al., 2018), and has additionally been shown to support *L. monocytogenes* growth, even at 4°C (Genigeorgis et al., 1991; Van Tassell et al., 2015; Holle et al., 2018).

Bacteriophage utilize a class of enzymes called endolysins to hydrolyze the host bacterium's peptidoglycan and complete their lytic cycle. Recently, these enzymes have been evaluated for use in several public health situations including food safety (Zhang et al., 2012), diagnostics (Schmelcher et al., 2010), disinfectants (Desbois et al., 2010), and as therapeutic agents (Loeffler et al., 2003; Cheng et al., 2005). Bacteriophages that infect gram-positive bacteria utilize modular endolysins composed of 2 clearly separated functional domains, the enzymatically active domain (**EAD**) and cell wall binding domain (**CBD**). The EAD is responsible for the enzymatic mechanism, and there are 5 primary classifications for EADs based upon the particular bond being hydrolyzed (Nelson et al., 2012). The CBD is responsible for binding to the bacterial cell wall and bringing the EAD to the vicinity of its substrate. Currently, *Listeria* bacteriophage endolysin CBDs have been categorized into 2 primary categories composed of 5 subclasses differing in binding properties such as serovars recognition, distribution on target bacterium, and binding affinity (Schmelcher et al., 2010); however, many remain to be categorized and assessed. In addition, in vitro evaluation of these endolysins has been shown to not fully predict efficacy upon practical application (Ibarra-Sánchez et al., 2018).

However, only a few studies have evaluated an endolysin's lytic efficacy as well as its implementation within a food system for addressing pathogen contamination. Previously, the endolysin PlyP100 was evaluated in a laboratory-scale fresh cheese model (Van Tassell et al., 2017). PlyP40 and PlyPSA are 2 of the only *Listeria* phage endolysins that have crystal structures (Korndörfer et al., 2006; Romero et al., 2018); however, their lytic activity has not been characterized nor has their implementation in a food matrix been evaluated. The objective of this study was to evaluate and compare the lytic activity of PlyP40 and PlyPSA under varying QF-relevant conditions in vitro and to implement these endolysins into a miniaturized laboratory-scale QF model and compare their efficacy with that of PlyP100.

Individual plasmids containing the gene sequences for PlyP100, PlyP40, and PlyPSA with an accompanying N-terminal His-tag were synthesized by Twist Biosciences (San Francisco, CA). The plasmids were then transformed into competent *Escherichia coli* BL21 (DE3). Respective cultures were grown aerobically with shaking (250 rpm) at 37°C to an optical density (**OD**) at 595 nm (OD_595_) of approximately 0.5 before induction with 400 µ*M* isopropyl-β-d-thiogalactoside. Cultures were then incubated for 24 h at 18°C, pelleted via centrifugation 4,000 × *g* for 15 min at 4°C, and washed in PBS (KCl, 200 mg/L; KH_2_PO_4_, 200 mg/L; NaCl, 8 g/L; Na_2_HPO_4_ 1.15 g/L, pH 7.2). Endolysins were purified from the cell pellets using the QIAexpress Ni-NTA Fast Start Kit (Qiagen, Valencia, CA) according to the manufacturer's instructions. Purified endolysins were dialyzed against PBS via Amicon Ultra-15 10 K Centrifugal Filter Unit (10 kDa molecular weight cutoff; Merck Millipore, Burlington, MA) before dilution with an equal volume of glycerol. Before storage (−20°C), the endolysins were sterilized via filtration. Purity and concentration were determined via 15% (wt/vol) SDS-PAGE and Quick Start Bradford Protein Assay (Bio-Rad Laboratories, Hercules, CA), respectively.

To determine the lytic spectrum of PlyP40 and PlyPSA, each endolysin was evaluated against the cellular debris of a broad collection of *Listeria* strains. The previously described PlyP100 was also analyzed. Included within the collection were several strains isolated from food outbreaks, including the cheese-relevant strain NRRL B-33104 derived from the Jalisco QF outbreak. Before each lytic assay, listerial strains (Table 1) were recovered from frozen glycerol stocks (−80°C) and cultured aerobically in brain-heart infusion broth (Becton, Dickinson and Co., Franklin Lakes, NJ) at 37°C. Cultures were heat-killed, centrifuged, and suspended in PBS. The cellular debris (OD_600_ = 1.0) was combined 1:1 in a microtiter plate with a final concentration of 2.5 µg of each endolysin. Cellular debris combined 1:1 with PBS served as a control in each experiment. The change in OD_600_ was measured over a 30-min period using a microplate reader (FilterMax F5 Multi-Mode Microplate Reader, Molecular Devices, San Jose, CA), held at 37°C.

**Table 1:** Activity (as shown by reduction in turbidity) of endolysins PlyP100, PlyP40, and PlyPSA against different *Listeria* species^1^

Species	Strain ID	Serovar	Reduction in turbidity (%)		
			PlyP100	PlyP40	PlyPSA
*L. monocytogenes*	10403S	1/2a	52.49 ± 0.30	36.12 ± 1.00	2.92 ± 0.93
	SLCC-5764	1/2a	33.87 ± 0.51	34.38 ± 0.73	11.86 ± 1.71
	NRRL B-33419^2^	1/2a	45.9 ± 0.60	38.68 ± 0.89	6.58 ± 1.40
	NRRL B-33395	1/2a	44.95 ± 0.37	40.72 ± 1.69	8.19 ± 1.89
	NRRL B-33391	1/2b	44.61 ± 0.43	29.07 ± 0.81	5.37 ± 0.62
	NRRL B-33424^2^	1/2b	47.32 ± 0.33	44.34 ± 1.00	4.96 ± 1.28
	ATCC 7644	1/2c	39.68 ± 0.12	32.62 ± 1.38	11.37 ± 0.62
	ATCC 19112	1/2c	40.9 ± 0.48	37.98 ± 0.72	5.71 ± 0.77
	NRRL B-33393	3b	44.42 ± 0.49	35.99 ± 2.13	7.89 ± 1.51
	NRRL B-33226	3c	42.29 ± 0.27	37.79 ± 0.99	4.60 ± 1.20
	NRRL B-33403	4a	48.88 ± 0.20	36.33 ± 1.69	35.98 ± 5.17
	ATCC 13932	4b	37.93 ± 0.68	28.95 ± 0.57	26.47 ± 2.00
	NRRL B-33420^2^	4b	41.44 ± 0.60	38.31 ± 1.00	20.78 ± 2.99
	NRRL B-33513^2^	4b	38.41 ± 0.29	17.67 ± 4.66	27.36 ± 0.79
	NRRL B-33104^2^	4b	48.58 ± 0.26	35.57 ± 0.95	23.23 ± 3.48
	NRRL B-33231	4c	41.10 ± 1.51	38.52 ± 0.99	31.17 ± 4.23
	NRRL B-33116	4d	39.32 ± 0.87	35.11 ± 1.27	15.95 ± 0.30
	NRRL B-33120	4e	37.76 ± 0.25	34.26 ± 1.36	25.82 ± 4.69
*L. innocua*	ATCC 33090	6a	44.85 ± 0.31	33.36 ± 2.45	23.01 ± 2.44
*L. ivanovii ssp. ivanovii*	NRRL B-33017	5	29.26 ± 1.26	35.03 ± 1.52	34.20 ± 0.04
*L. seeligeri*	NRRL B-33019	1/2b	49.16 ± 0.49	38.31 ± 2.18	14.67 ± 1.90

1 Values are means ± SEM for triplicate independent experiments.

2 Isolated strains associated with food outbreaks.

All *L. monocytogenes* strains, spanning multiple serovars, were lysed by PlyP100, PlyP40, and PlyPSA. Whereas PlyP100 showed the greatest activity across all of the strains, PlyPSA showed the least (Table 1). For 10 of the 21 strains (48%), PlyPSA was able to reduce the turbidity by more than 15%. The greatest reductions for PlyPSA were observed against *L. monocytogenes* serovar group 4. Comparatively, however, PlyP100 was able to show at least a 29.26% ± 1.26% reduction against all strains, with the highest observed activity against the laboratory strain 10403S of *L. monocytogenes* (serovar 1/2a). Although PlyP40 showed a comparable range of reductions to that of PlyP100, its highest activity only resulted in a 44.34% ± 1.00% reduction over 30 min. All 3 endolysins also showed activity against *Listeria innocua, Listeria ivanovii*, and *Listeria seeligeri*.

Although activity of PlyPSA was lower than that of PlyP40 and PlyP100, it was still active against all tested strains. However, it showed lesser activity against *Listeria* 1/2 and 3 serovars. This suggests that PlyPSA may not be a strong candidate for food safety use, because *L. monocytogenes* serovars 1/2a and 1/2b are the 2 serovars most commonly implicated in human foodborne illness (Kathariou, 2002). The lytic spectrum for both PlyPSA and PlyP40 has not been extensively evaluated before this study; however, the binding spectrums for the CBD of both PlyPSA and PlyP40 alone corroborate their lytic spectrums (Schmelcher et al., 2010). Previously, Schmelcher et al. (2010) showed that PlyP40 binds strongly to all serovars of *Listeria*, whereas PlyPSA showed little to no binding for serovars 1/2a, 1/2b, 3a, 4b, 3c, and 7.

Endolysin activity is modulated by key, food-relevant environmental factors such as pH, NaCl concentration, and temperature. To determine how the lytic activity of each endolysin would be affected by these environmental factors, 2.5 µg of each endolysin was tested against *L. innocua* (ATCC 33090) cellular debris under varying QF-relevant pH values (6–8), NaCl concentrations (0.0–2.5%), and temperatures (4, 25, and 37°C; Ibarra-Sánchez et al., 2017; Holle et al., 2018), as previously described (Van Tassell et al., 2017). Briefly, for pH and salt lytic assays, 40 m*M* boric acid/phosphoric acid buffer (**BP**) was used in place of PBS buffer and adjusted to the corresponding pH levels (6–8). The NaCl experiments also used BP buffer (pH 7) with different amounts of NaCl dissolved therein (0–2.5% wt/vol). To evaluate the effect of temperature on the lytic activity of the endolysins, all assay materials were incubated at different temperatures (4, 25, or 37°C).

The effect of pH and salt concentration on lytic activity of each endolysin was evaluated individually within QF-appropriate ranges (pH 6–8, NaCl 1.0–2.5%; Figure 1). The lytic activity of each endolysin was evaluated in BP buffer over a pH range from 6.0 to 8.0. Both PlyP100 and PlyP40 showed the greatest activity around pH 7. As acidity increased, however, PlyP100 activity decreased, whereas that of PlyP40 did not. At the other end of the spectrum, activity of PlyPSA was highest at pH 8 and showed an approximately 50% decrease at pH 7. As the acidity increased to pH 6, activity of PlyPSA continued to decrease. The effect of NaCl on the lytic activity of endolysins was also evaluated within the commonly observed salt concentrations for QF (Holle et al., 2018). All endolysins showed activity within the common QF salt range of 1 to 2%. PlyP100 maintained its highest activity between 1.5 and 2.0% NaCl (wt%), which is the typical range for QF (Holle et al., 2018). As the salt concentration increased, lytic activity of PlyP40 began to decrease, with approximately one-tenth of its activity remaining at 2.5% NaCl. In contrast, the activity of PlyPSA decreased when the salt concentration was <1% or >2%.

**Figure 1:**
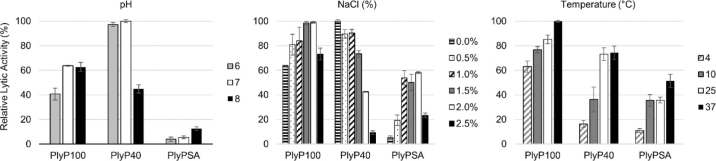
Optimal queso fresco-relevant environmental conditions for the lytic activity of endolysins PlyP100, PlyP40, and PlyPSA. The optimal (A) pH, (B) NaCl, and (C) temperature conditions for the lysis of heat-killed *Listeria innocua* ATCC 33090 was characterized via turbidity reduction assay over 30 min using 2.5 µg/mL concentrations of each endolysin. The highest reduction value across all 3 endolysins was set to 100% for comparison. Bars indicate the mean ± SEM for triplicate independent experiments.

We evaluated the long-term stability of the endolysins to evaluate their shelf life stability. Each endolysin (250 µg) was held at 4 and 25°C in PBS for 28 d while evaluating its activity every 7 d, as previously described (Loeffler et al., 2003). At each sampling point, the lytic activity against *L. innocua* (ATCC 33090) debris was measured as described above. We observed no decrease in lytic activity among any of the endolysins (data not shown).

Food matrices are complex beyond the simple parameters evaluated in vitro; thus, each endolysin was incorporated into a fresh cheese model inoculated with *L. monocytogenes* (NRRL B-33104). The cheeses were prepared as previously described (Van Tassell et al., 2017), and PlyP40, PlyPSA, and PlyP100 were each added to the drained curd of their respective cheeses at a final concentration of 250 µg/g of cheese. Data were analyzed by one-way ANOVA, and the antimicrobial effect compared with the control was evaluated using Tukey's test to determine statistical significance (α < 0.05) of mean differences using the data analysis add-on in Microsoft Excel (Redmond, WA). Asterisks indicate significant difference compared with the control.

The *L. monocytogenes* counts from the untreated miniaturized laboratory queso fresco (MLQF)showed growth of approximately 4 log cfu/g after 28 d with a final level of ~8.6 log cfu/g (Figure 2). PlyPSA hindered the growth of *L. monocytogenes* in the MLQF; however, after 28 d, *Listeria* counts had increased to 8.5 log cfu/g, which was not significantly (α < 0.05) different from the control. PlyP40 showed a similar effect to PlyPSA, but was more effective, reducing the viable cell counts to only 7.9 log cfu/g. PlyP100 was the most effective, at the evaluated concentration, at limiting cell counts of the inoculum.

**Figure 2:**
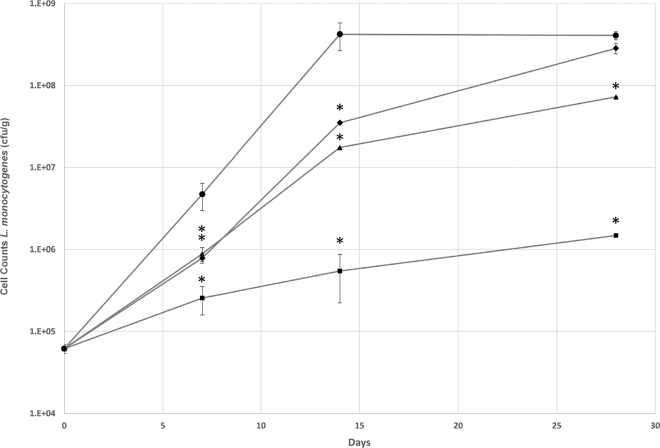
Antimicrobial activity of endolysins PlyP100 (□), PlyP40 (δ), and PlyPSA (♦), compared with a PBS control (•) against *Listeria monocytogenes* NRRL B-33104 in a miniaturized laboratory-scale queso fresco over 28 d of storage at 4°C. Each endolysin was added at 250 µg/g of cheese. Values are means ± SEM for triplicate independent experiments. One-way ANOVA was performed, and the antimicrobial effect compared with the control was evaluated using Tukey's test to determine statistical significance (α < 0.05) of mean differences. Asterisks indicate significant difference (*P* < 0.05) compared with the control.

In summary, we evaluated the lytic spectrums for PlyP40 and PlyPSA and both were capable of lysing the lytic debris from all of the tested *Listeria* strains. The evaluated endolysins all maintained optimal activity at 37°C; however, the degree of activity lost at colder temperatures varied among the enzymes. Similar to that of PlyP100, the activity of PlyPSA increased as pH increased, whereas PlyP40 exhibited the opposite behavior. PlyP40 was the most effective enzyme at lower salt concentrations, suggesting that there may be situations in which it is the optimal *Listeria* phage endolysin compared with PlyP100 and PlyPSA. PlyP100 was still the most effective endolysin at controlling *L. monocytogenes* in QF. Future work could focus on further evaluating situations in which PlyP40 may be an optimal endolysin. Additionally, further insight into the functional capacity of PlyP100 EAD and CBD should elucidate the observed effectiveness. Genetic manipulations (e.g., domain swapping) may further increase our understanding of these endolysins and increase their ability to be applied for food safety applications.
